# Application of Endogenous Stem Cells in the Repair of Annulus Fibrosus Injury of Intervertebral Discs

**DOI:** 10.1155/sci/9974294

**Published:** 2025-10-21

**Authors:** Wenxuan Zhao, Yuang Zhang, Bin Han, Han Zhou, Qixin Chen

**Affiliations:** ^1^The Department of Orthopedics, The Second Affiliated Hospital of Zhejiang University School of Medicine, Hangzhou, Zhejiang Province, China; ^2^The Department of Rehabilitation Medicine, The Second Hospital of Zhejiang University School of Medicine, Hangzhou, Zhejiang Province, China; ^3^Orthopedics Research Institute of Zhejiang University, Hangzhou, Zhejiang Province, China; ^4^Key Laboratory of Motor System Disease Research and Precision Therapy of Zhejiang Province, Hangzhou, Zhejiang Province, China; ^5^Clinical Research Center of Motor System Disease of Zhejiang Province, Hangzhou City, Zhejiang Province, China

**Keywords:** annulus fibrosus repair, endogenous stem cells, intervertebral disc degeneration, microenvironment, regenerative medicine

## Abstract

Intervertebral disc degeneration (IVDD), a major contributor to chronic low back pain (LBP), involves progressive extracellular matrix (ECM) degradation and limited self-repair. Current therapies alleviate symptoms but fail to halt degeneration, driving interest in endogenous stem cell-based regeneration. Endogenous stem/progenitor cells within disc niches exhibit regenerative potential through ECM synthesis, anti-inflammatory signaling, and exosomal miRNA-mediated repair. Preclinical studies highlight mesenchymal stem cell (MSC) transplantation and reprogramed induced pluripotent stem cells (iPSCs) in restoring disc hydration and reducing pain, while early clinical trials report symptomatic relief (e.g., 70% pain reduction) but incomplete structural recovery. Challenges include the disc's hostile microenvironment (hypoxia and nutrient deprivation), age-related depletion of endogenous stem/progenitor cells, and impaired cell homing under mechanical stress. Emerging strategies target epigenetic modulation, biomimetic scaffolds, and combination therapies to enhance cell survival and integration. Despite promising preclinical outcomes, clinical translation requires overcoming microenvironmental barriers and refining delivery systems. Future efforts should prioritize large-animal validation and biomarker-guided approaches to bridge the gap between experimental success and therapeutic application.

## 1. Introduction

A global study assessing the incidence, prevalence, and years lived with disability (YLDs) of 354 diseases across 195 countries identified low back pain (LBP) as the leading cause of productivity loss (measured by YLDs) and the primary disabling health condition in 126 nations [1]. Based on its pathogenesis, LBP can be classified as mechanical, neuropathic, or primarily nociplastic. The prevalence of neuropathic pain in chronic LBP patients ranges from 16% to 55% [2]. Current evidence suggests that neuropathic pain is predominantly associated with intervertebral disc herniation and spinal stenosis, with disc herniation being the most common cause of radicular pain [1]. However, degenerated discs exhibit limited self-repair capacity. Traditional therapies, such as pharmacologic analgesia and surgical fusion, alleviate symptoms but fail to reverse degeneration and may even accelerate adjacent segment degeneration. Consequently, regenerative strategies centered on endogenous stem cells (which are naturally present in the body and possess self-renewal and differentiation potential, in contrast to exogenous stem cells that are obtained through laboratory culture or other artificial methods and transplanted into the body) have emerged as a research focus. This review outlines the pathophysiology of intervertebral disc degeneration (IVDD), summarizes advances in annulus fibrosus (AF) repair using endogenous stem cells, and discusses current challenges and future directions.

## 2. Pathophysiology of IVDD

Anatomically, the intervertebral disc (IVD) comprises the nucleus pulposus (NP), AF, and cartilaginous endplate (CEP) [3]. The IVD functions as a shock absorber, distributing mechanical loads, and enabling spinal mobility [4]. As the IVD bears the weight of the torso and upper limbs while simultaneously withstanding significant shear forces during spinal movement, it is particularly susceptible to mechanical wear and degeneration in daily activities and occupational labor. Furthermore, being the largest avascular organ in the human body [5], the disc relies predominantly on nutrient diffusion through the cartilage endplate for sustenance, with only minimal direct blood supply. This unique anatomical characteristic renders the IVD highly vulnerable to degenerative changes.

The structural integrity of the IVD depends on its extracellular matrix (ECM), composed of collagen, proteoglycans, elastin, and water. The NP, rich in type II collagen, proteoglycans, and water, provides compressibility, while the AF, composed of type I collagen with lower hydration, confers tensile strength [6]. The AF surrounds the central NP, which is mechanically restrained to prevent the NP from protruding outward. An intact AF maintains the height and shape of the IVDs, ensuring the normal biomechanical function of the spine. At the same time, the fiber arrangement of the fiber ring can withstand stresses in multiple directions (e.g., compression, shear, and torsion). Its integrity ensures that the load is evenly distributed to the endplate and surrounding bone tissue during spinal movement, avoiding local stress concentrations. Rupture or degeneration of AF is a key trigger for a herniated disc. Trauma or overloading can lead to AF fiber tears, causing acute flank pain and limited mobility. Chronic damage to AF (e.g., age-related collagen degradation and repeated microinjury) leads to the release of inflammatory factors (e.g., IL-1β and TNF-α), matrix-degrading enzymes (e.g., MMPs) and the degradation of cartilage oligomeric matrix protein (COMP) [7], which accelerates the decomposition of IVD stroma (e.g., proteoglycans and collagen), increases type I collagen and decreases type II collagen, while the water content of AF and NP gradually decreases, the NP loses its elasticity, and cracks appear in the AF. Therefore, under the accumulation of strain or external force, the partial or total rupture of the AF, leading to the protrusion of the AF, and even the NP and cartilage endplate to the back, and in severe cases, the symptoms of nerve compression appear, and finally the degenerative disc disease (DDD) is triggered.

In conclusion, the IVD's susceptibility to degeneration is primarily due to its unique anatomy, including limited blood supply, reliance on nutrient diffusion, and the mechanical stresses it endures, with degeneration of the AF playing a critical role in the onset of disc herniation and DDD.

## 3. Comparative Analysis of Current Therapeutic Strategies

Currently, widely used treatment options for lumbar disc herniation can be primarily categorized into conservative treatment and surgical treatment. Conservative treatment mainly includes medication, physical therapy, and comprehensive traditional Chinese medicine therapies. The advantage of medication lies in its ability to alleviate pain symptoms in the short term, with minimal damage and side effects to patients. However, medication is suitable for patients with mild to moderate AF injuries and has limited efficacy for severe AF ruptures. Long-term use of nonsteroidal anti-inflammatory drugs (NSAIDs) may also increase gastrointestinal, liver, and kidney damage. Physical therapies such as traction, massage, heat therapy, shockwave therapy, and interferential current therapy can help alleviate muscle spasms and improve local blood circulation, providing short-term relief for mild to moderate pain [1, 8]. However, the reduction in pain is generally modest, and long-term follow-up of patients shows no significant benefits in terms of pain reduction or disability improvement [9]. Surgical treatment can be divided into minimally invasive surgery and open surgery. Minimally invasive techniques include, for example, percutaneous endoscopic lumbar discectomy (PELD/PTED), unilateral biportal endoscopic spinal surgery (UBE), and arthroscope-assisted uniportal spinal endoscopic surgery (AUSS). Open surgery involves traditional procedures such as simple discectomy and posterior lumbar decompression with bone grafting, fusion, and internal fixation. The advantage of surgical treatment is that it can directly remove the herniated disc and nerve-compressing tissues, providing significant postoperative pain relief. However, surgical treatment is more invasive, and the removal of the affected IVD disrupts the original biomechanical function of the spine, accelerating degeneration and damage in adjacent segments.

It is evident that traditional treatment approaches (medication, physical therapy, and surgery) have certain limitations in repairing damaged AF and restoring the biomechanical function of IVDs. Stem cell-based regeneration of the AF has emerged as a prominent research focus. Since the 21^st^ century, cell-based transplantation therapies and tissue bioengineering have been applied to tissue regeneration and have become potential treatments for IVDD. Animal studies have demonstrated the efficacy of cell transplantation therapy for IVDD, utilizing NP cells (NPCs), bone marrow-derived mesenchymal stem cells (BMSCs), olfactory mucosa-derived mesenchymal stem cells (OMSCs), adipose-derived mesenchymal stem cells (AMSCs), and induced pluripotent stem cells (iPSCs) as cellular “seeds” for transplantation [10]. However, the harsh microenvironment of the IVD poses significant challenges to cell-based IVDD therapies [11]. The disc's high osmotic pressure, mechanical loading, nutrient deficiency, low oxygen tension, and acidic pH collectively impair the viability, proliferation, and differentiation of transplanted cells [12–14]. Compared to exogenous stem cell transplantation, endogenous stem cells offer several advantages, including reduced immune rejection and better adaptation to the degenerative disc microenvironment. For example, endogenous NP mesenchymal stem cells (NPMSCs) may better tolerate the hyperosmotic and acidic environment of the IVD compared to AMSCs [14–16], which could be related to the expression of acid-sensing ion channel 3 [17, 18]. However, the specific mechanisms require further investigation. As a result, endogenous stem cell-based approaches have become a leading research direction in this field.

In conclusion, traditional treatment options (medication, physical therapy, and surgery) and exogenous stem cell therapies have certain limitations, whereas the numerous advantages of endogenous stem cell therapies have positioned them as the current mainstream research direction.

## 4. Distribution and Types of Endogenous Stem Cells

In recent years, a growing body of research has identified endogenous cells with stem/progenitor cell characteristics in the IVDs and within NP tissues (Table 1) [19, 25, 28, 29, 31–40]. In the 1970s, Schofield first proposed the concept of the stem cell niche (SCN) [41], defined as a dynamic microenvironment composed of ECM and neighboring cells that regulate resident stem cells [27, 41–45]. The migration of endogenous stem cells from their niche to adjacent target regions is a critical process in tissue self-repair. Over the past decade, researchers have hypothesized the existence of a potential IVD SCN (ISN) within the disc region [28], which may play a significant role in IVD regeneration. Accordingly, based on anatomical distinctions, IVD progenitor cells (IVDPCs) are currently classified into three subpopulations: NP-derived progenitor cells (NPPCs), AF-derived progenitor cells (AFSPCs), and CEP-derived progenitor cells (CEPCs) [46].

Risbud et al. [47] were the first to identify a cell population in the human AF region expressing stem cell surface markers and exhibiting multilineage differentiation potential. Henriksson et al.[27, 28] further supported this by using 5-bromo-2′-deoxyuridine (BrdU) labeling to detect slow-cycling cells (a hallmark of stem cells) in rabbit models, suggesting that the ISN may reside at the AF-ligament junction and perichondrial regions.

Sakai et al.[22] systematically characterized molecular markers of NP progenitor cells (NPPCs) through cross-species studies. Their work demonstrated that Tie2^+^ and GD2^+^ dual-positive cells exhibit stem/progenitor properties in both murine and human samples, capable of forming spheroid colonies expressing collagen II and aggrecan, differentiating into mesenchymal lineages, and promoting NP tissue regeneration. Johnson et al.[48] further demonstrated that human IVD aggrecan inhibits nerve growth and repels sensory nerves, as an important pathogenetically significant component of pain formation. This may contribute to the alleviation of “discogenic” LBP triggered by the induction of neoinnervation [48, 49]. Erwin et al. [26] also confirmed the presence of NPPCs expressing classical MSC genes but exhibiting broader differentiation plasticity than conventional MSCs.

Kim et al.[50, 51] revealed that rabbit notochordal cells could stimulate CEP-derived cell migration into the NP region, facilitating the transition from notochordal NP to fibrocartilaginous NP. Similarly, Liu et al. [29] and Xiong et al. [52] independently isolated and characterized CEP-derived stem cells (CEPSCs) with migratory potential and enhanced osteochondrogenic differentiation capacity in vitro.

Previous studies have demonstrated that IVDPCs exhibit surface markers (CD29, CD44, CD73, CD90, and CD105) consistent with BMSCs [33, 53], while lacking hematopoietic lineage markers (CD34, CD45, CD14, CD11b, CD79, and CD19) and HLA-DR expression [20, 21, 23–25, 54, 55]. Notably, IVD stem/progenitor cells (IVDSPCs) additionally express certain markers shared by other stem/progenitor cell populations [33, 38, 56, 57]. Importantly, research has identified unique surface markers distinguishing IVDPCs from other stem cells or resident IVD cells. A seminal discovery by Sakai's team revealed that tyrosine kinase endothelial receptor (Tie2) and disialoganglioside 2 (GD2) serve as specific markers for NPPCs [22]. There is currently limited research on the comparison of specific surface marker expression among different subgroups of IVDPCs [58]. However, based on existing studies, it is generally believed that CD44 serves as a specific marker for NPPCs [59]. As CD44 is a cell surface receptor for hyaluronic acid, it may provide a better explanation for the higher content of hyaluronic acid in the NP compared to other parts of the IVD. This may also help explain why the injection of hyaluronic acid gel can promote NP repair but fails to repair the damaged AF [60].

The advent of single-cell sequencing technologies promises to revolutionize the precise classification of IVD cellular subpopulations, offering unprecedented resolution in disc cell biology research. Wang et al. [61] utilized RNA sequencing to classify IVD cells into five distinct clusters. These included NPCs (expressing CD24, ANXA3, and KRT19) and outer AF/inner AF (OAF/IAF) cells (expressing ACAN, COL1A1, and COL2A1). Two additional clusters were designated as a stem-like cluster and another cell cluster based on their highly expressed genes. The study confirmed the presence of a heterogeneous cell population within the IVD exhibiting stem cell differentiation potential. This population expressed specific marker genes, including but not limited to Eng, MCAM, and Tie-related genes, and demonstrated utility for in vitro chondrogenic and osteogenic differentiation. Li et al.'s [62] research classified the cells in the CEP region into mesenchymal chondrocytes, NP mesenchymal stem/progenitor cell (NPMSC or NPPC), stromal, smooth muscle/pericyte, blood and endothelial cells. Among them, NPMSC cells highly specifically expressed the PDGFRA and PRRX1 genes. Meanwhile, they further divided the mesenchymal chondrocytes into three cell clusters. However, all chondrocytes highly expressed MSC marker genes APOD, DCN, and MGP. Therefore, these chondrocytes were regarded as subpopulations with potential differentiation functions.

In conclusion, recent research has identified distinct populations of endogenous progenitor cells within the IVD, with specific molecular markers and differentiation potential, highlighting the existence of a SCN that may play a crucial role in IVD regeneration and repair.

## 5. Mechanism of Action of Endogenous Stem Cells in Annulus Repair of IVDs

Research indicates that during AF degeneration or injury, degenerated IVD tissues locally release inflammatory factors (e.g., TNF-α and IL-1β), chemokines (e.g., SDF-1), and matrix degradation products (e.g., hyaluronan fragments). These mediators activate endogenous stem cell migration signaling pathways (such as the CXCR4/SDF-1 axis) to recruit stem cells. The principal chemotactic factors involved include: transforming growth factor-beta (TGF-β), stromal cell-derived factor-1 (SDF-1/CXCL12), insulin-like growth factor-1 (IGF-1), C–C motif chemokine ligand 5 (CCL5/RANTES) [63–66], and so on. Concurrently, hypoxia-inducible factor 1-alpha (HIF-1α) and mechanical stress alterations promote the directional migration of stem cells to injury sites by modulating critical signaling pathways including Notch and Wnt. It has been demonstrated that the migration of MSCs to hypoxic areas increases the production of various therapeutic paracrine mediators [67]. For example, bone marrow (BM) -derived MSCs exposed to hypoxia for 24 h (1% oxygen) induce the production of vascular endothelial growth factor (VEGF), fibroblast growth factor 2 (FGF-2), hepatocyte growth factor (HGF), and IGF-1 in an NF-κB-dependent manner [68].

Studies have shown that endogenous stem cells (potentially originating from the NP, AF, or articular cartilage endplate areas) differentiate into AF-like cells under the induction of the local microenvironment (e.g., growth factors such as TGF-β and BMPs), replenishing lost functional cells. The differentiated stem cells secrete growth factors (e.g., TGF-β and IGF-1) and anti-inflammatory cytokines to regulate the synthesis of ECM (e.g., key matrix components like collagen II and aggrecan) [69–72], thereby restoring the structural integrity of the AF. In addition, the hypoxic environment leads to the activation of HIF-1α, which subsequently induces the transcription of angiogenic genes, such as VEGF [73, 74], as well as the MSC chemotactic factor SDF-1 [75]. By releasing anti-inflammatory factors, such as IL-10, the release of pro-inflammatory factors (IL-6, IL-1β, and inducible nitric oxide synthase [iNOS]) is reduced, and the polarization of M1 macrophages to M2 macrophages is induced [76], thereby alleviating cell necrosis and apoptosis caused by inflammation.

**Figure 1:**
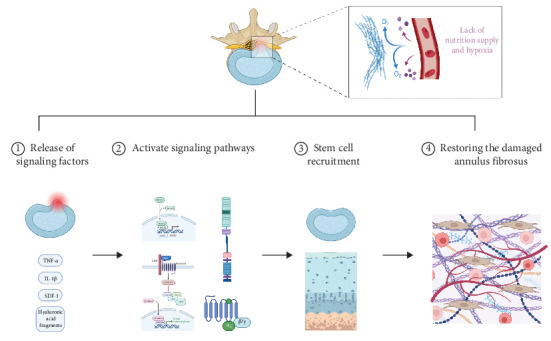
Mechanism of action of endogenous stem cells in annulus repair of intervertebral discs.

In the hypoxic and nutrient-deficient microenvironment of the IVD, stem cells enhance their glycolytic capacity to sustain survival while mitigating oxidative stress damage through ROS regulation. It has been demonstrated in vitro that exposure of BM-MSCs to hypoxia results in increased cell proliferation and the expression of stemness markers such as Rex-1 and Oct-4, indicating an enhancement of stemness in BM-MSCs under hypoxic conditions [74]. They modulate the reparative behavior of target cells by delivering miRNAs (e.g., miR-221 and miR-146a) and functional proteins via exosomes [77]. It is possible that miRNAs influence the ectopic expression of Oct4, Sox2, Klf4, and c-Myc (Yamanaka factors/OSKM), thereby reprograming differentiated cells to a pluripotent state [77, 78]. Additionally, stem cells upregulate collagen and proteoglycan synthesis-related genes (e.g., SOX9 and ACAN) to promote matrix deposition, while downregulating matrix metalloproteinases (MMP-3 MMP-13) and ADAMTS family expression to reduce ECM degradation, thereby orchestrating ECM remodeling (Figure 1) [15, 63, 79, 80].

In conclusion, endogenous stem cells, particularly under the influence of the hypoxic, inflammatory, and nutrient-deficient microenvironment of the IVD, enhance their regenerative potential by migrating to injury sites, secreting therapeutic paracrine factors, and promoting ECM remodeling, thereby contributing to tissue repair and inflammation resolution.

## 6. Progress in Preclinical and Clinical Research

The aforementioned research not only confirms the isolation and identification of cells with stem/progenitor characteristics in all IVD components (NP, AF, and CEP), but more critically, demonstrates their migratory capacity from ISNs to degenerated NP, AF, and CEP tissues. Consequently, the cornerstone of endogenous repair strategies lies in two synergistic approaches: augmenting the quantity and functional viability of IVDSPCs within degenerated discs and stimulating their targeted migration from niche reservoirs to sites of degeneration.

To augment the quantity and viability of IVDSPCs in degenerated IVDs, a straightforward strategy involves direct supplementation of endogenous stem/progenitor cells. Although challenges persist in isolating endogenous IVDSPCs, numerous studies have identified surface marker similarities between IVDSPCs and BM-derived MSCs, positioning endogenous MSCs as promising alternatives. Preclinical and clinical investigations demonstrate that MSC injection significantly mitigates disc degeneration [10, 81]. Wang et al. [82] reported that injectable hydrogel-loaded NP-MSC transplantation delays degeneration and promotes regeneration in rat models. Pereira et al. [83] found MSC-seeded cartilage endplates effectively remodel ECM in degenerated discs. Hu et al. [84] developed a novel melatonin-loaded dynamic hydrogel, which successfully repaired the box-cut defect in the AF tissue of rat tails. In clinical translation, Phase I/II trials (NCT01513694) revealed tricalcium phosphate scaffolds combined with MSCs achieve 80% lumbar fusion rates without severe adverse events [85]. Preliminary clinical trials of autologous BM-MSC injections showed 70% pain reduction (visual analog scale, VAS) and 35% MRI T2-signal improvement at 12 months, though disc height restoration remained below 15% [86]. A 24-month follow-up study demonstrated sustained functional improvement (Oswestry Disability Index, ODI) in female patients treated with collagen sponge-loaded BM-MSCs, suggesting mechanisms potentially involving ECM hydration modulation rather than structural reconstruction [87].

In summary, enhancing the quantity and functionality of IVDSPCs through direct supplementation, alongside stimulating their migration to degenerated sites, holds promise for repairing disc degeneration, with preclinical and clinical studies showing positive outcomes, particularly with MSC-based therapies.

## 7. Challenges and Future Directions

Nevertheless, stem cells within niche reservoirs and recruited IVDSPCs remain vulnerable to the hostile microenvironment of degenerated discs (e.g., mechanical compression and hypoxia), frequently undergoing excessive cell death that critically compromises their survival and regenerative capacity [12–14] This pathological cell attrition constitutes a fundamental barrier to maintaining viable IVDSPC populations. Mechanistic studies reveal that under such adverse conditions, NPCs and other IVD components activate multiple interlinked cell death pathways including caspase-dependent classical apoptosis [88], JNK-mediated stress-induced apoptosis [89], BNIP3/NIX-driven mitophagy [89], PI3K/AKT pathway modulation [90], RIPK1/RIPK3/MLKL-regulated necroptosis [91], and the cross-communication between them lead to cell death apoptosis, autophagy, or necroptosis.

The progressive age-related decline in stem/progenitor cell populations within the IVD microenvironment represents a critical challenge for endogenous repair mechanisms. Substantial evidence from recent studies demonstrates a significant inverse correlation between the quantity of IVD-resident stem/progenitor cells and both advancing age and degenerative severity [22, 92, 93]. Notably, comparative analyses reveal that NPPCs from aged individuals exhibit characteristic senescent phenotypes—including reduced proliferative capacity, mitochondrial dysfunction, and replicative exhaustion—when contrasted with their younger counterparts [25]. These findings collectively indicate that age-associated depletion of functional stem/progenitor cell pools, particularly the accelerated cellular senescence signatures observed in degenerative contexts, constitutes a pivotal pathological factor undermining the IVD's intrinsic regenerative capacity.

The progressive decline in chemotactic migratory capacity of stem/progenitor cells within the IVD microenvironment represents another critical challenge for endogenous repair strategies. Research demonstrates that stem cell migration exhibits distinct microenvironment-dependent characteristics [27, 94, 95]. Under mildly degenerative conditions (2.2% O_2_), MSCs significantly enhance their proliferative activity and directional migration through activation of the HIF-1α/FASN/mTORC1 signaling axis [95]. Conversely, in pathologically hypoxic environments (1% O_2_), MSCs experience drastic impairment in trans-endothelial migration capacity via GTPase RhoA activity modulation [94]. Furthermore, mechanical stress studies reveal increased stem cell recruitment at moderate loading (19.6 N) versus significant reduction under excessive compression (78.4 N). These findings collectively indicate that extreme microenvironmental insults may overwhelm endogenous repair systems through compensatory deactivation of cell migration-homing mechanisms, ultimately leading to systemic failure.

In addition, cryopreservation of endogenous stem cells remains a challenge. Traditionally, cryopreservation protocols use high concentrations of cryoprotectants, such as dimethyl sulfoxide (DMSO). On one hand, some complications associated with the use of these permeable cryoprotectants include an exceptionally low glass transition temperature [96], which may reduce the viability of cryopreserved cells. More importantly, cryopreservation media containing DMSO have been shown to be toxic to patients [97, 98]. Therefore, it is necessary to explore a novel, safe, and nontoxic preservation method that can maintain stem cell viability as much as possible. For example, Buchanan et al. [99] found that loading stable trehalose into hematopoietic stem and progenitor cells (HPCs) allowed them to be stored at ambient temperature (25°C) for 4 weeks while still maintaining high activity.

Future research on endogenous stem cell-mediated repair of degenerated IVD AF should focus on precisely identifying stem cell sources within their niches and analyzing their dynamic changes in degenerative microenvironments. This requires developing molecular agents to regulate key signaling pathways controlling proliferation and migration, thereby enhancing endogenous stem cell recruitment and differentiation. For example, current leading candidate factors for mobilizing endogenous stem cells to repair IVD tissue include BMP13 or GDF6 [100–104], as well as potential drugs regulating inflammation in disc degeneration, such as CGS-21680 (an A2AR agonist) [105]. Concurrently, investigations should explore epigenetic reprograming mechanisms (e.g., DNA methylation, histone modifications, and noncoding RNAs) to counteract stem cell aging, while targeting antiaging pathways involving telomerase activity, mitochondrial function, and autophagy to preserve cellular vitality. Advanced biocompatible scaffolds with oxygen self-supply and mechanical adaptability must be engineered to optimize local microenvironments. For example, Zheng et al. [106] developed a PFTBA core–shell oxygen supply scaffold, which can protect AFSCs from hypoxia-induced cell death through the localized release of oxygen, while promoting cell migration and ECM production. Large animal model validation, multimodal combination therapies (gene/physical interventions), and personalized precision strategies (biomarker screening and image-guided delivery) should be systematically developed. Ultimately, interdisciplinary integration will address critical challenges in stem cell homing, survival, and functional integration, supported by ethical frameworks to accelerate the translation of foundational discoveries into clinical applications for structural and functional disc restoration.

In conclusion, the challenges to endogenous stem cell-mediated repair of degenerated IVDs include cell death due to the hostile microenvironment, age-related decline in stem/progenitor cell function, impaired migratory capacity, cryopreservation issues, and the need for targeted strategies to enhance stem cell recruitment, survival, and differentiation through molecular agents, epigenetic reprograming, and advanced biomaterial scaffolds.

## 8. Conclusion

In summary, endogenous stem cell populations within the IVD—such as NP-derived stem cells (NPSCs) and cartilage endplate-derived stem cells (CESCs)—possess unique microenvironmental adaptability and multipotent differentiation potential. They promote the repair of degenerated discs through mechanisms, such as paracrine signaling, ECM remodeling, and immunomodulation. Numerous preclinical studies and early clinical trials support the therapeutic potential of endogenous stem cells in IVD regeneration.

However, this field still faces critical challenges, including low stem cell homing efficiency, interference from microenvironmental inhibitory factors, and issues related to stem cell senescence and death. Endogenous stem cell therapy represents a highly promising biological strategy for disc repair, but its successful clinical translation depends on deep collaborative innovation between fundamental research and clinical practice.

## Figures and Tables

**Table 1:** Species and surface markers of IVDSPCs.

Cell type	Species	Surface markers	References
NPSPCs	Human	CD73^+^, CD90^+^, CD105^+^, CD34^−^, CD45^−^, HLA-DR^−^	Liu et al. [12]
	Human	CD29^+^, CD44^+^, CD105^+^, CD14^−^, CD34^−^, CD45^−^, HLA-DR^−^	Shen et al. [19]
	Human	CD73^+^, CD90^+^, CD105^+^, CD29^−^, CD45^−^	Qi et al. [20]
	Human	CD73^+^, CD90^+^, CD105^+^, CD34^−^, CD45^−^	Chen et al. [21]
	Human	Tie2^+^, GD2^+^, Flt1^+^, CD271^+^, CD24^−^	Sakai et al. [22]
	Human	CD90^+^, CD73^+^, CD105^+^, CD106^+^, CD14^−^, CD19^−^, CD24^−^, CD45^−^, HLA-DR^−^	Blanco et al. [23]
	Human	Tie2^+^, GD2^+^, Oct-4^+^, Sox-2^+^	Li et al. [24]
	Rat	CD73^+^, CD90^+^, CD105^+^, CD34^−^, CD45^−^	Zhao et al. [25]
	Canine	CD133^+^, KI67^+^, Nanog^+^, NCAM^+^, Oct3/4^+^, Sox2^+^,	Erwin et al. [26]

AFSPCs	Human, rat, rabbit	Notch1^+^, Delta4^+^, CD117^+^, STRO-1^+^, C-kit low, KI67 low	Henriksson et al. [27]
			Henriksson et al. [28]

CEPSCs	Human	CD44^+^, CD73^+^, CD90^+^, CD105^+^, CD133^+^, CD14^−^, CD19^−^, CD34^−^, CD45^−^, HLA-DR^−^	Liu et al. [29]
	Human	CD73^+^, CD90^+^, CD105^+^, CD34^−^, CD45^−^, HLA-DR^−^	Yuan et al. [30]

## Data Availability

The data sharing is not applicable to this article, as no new data were created or analyzed in this study.
